# Cervicothoracic Tuberculous Spondylitis in a Toddler: A Case Report

**DOI:** 10.1055/a-2952-7393

**Published:** 2026-09-18

**Authors:** Paul van Schie, Bregje Jaeger, Martijn van der Kuip, Agnita Stadhouder, Ricardo E. Feller

**Affiliations:** 1 Department of Neurosurgery 26066 Amsterdam University Medical CenterAmsterdamThe Netherlands; 2 Department of Child Neurology 26066 Amsterdam University Medical CenterAmsterdamThe Netherlands; 3 Department of Pediatric Infectious Diseases and Immunology 26066 Amsterdam University Medical Center, Emma Children’s HospitalAmsterdamThe Netherlands; 4 Department of Orthopedic Surgery and Sports Medicine 26066 Amsterdam University Medical CenterAmsterdamThe Netherlands

**Keywords:** tuberculosis, cervicothoracic spine, toddler, sternotomy

## Abstract

**Background and Importance** Tuberculous spondylitis (or Pott’s disease) is a common form of skeletal tuberculosis. Although primarily treated with tuberculostatic drugs, spinal surgery is necessary in patients with deformities or spinal cord compression. Literature describing surgery strategies in very young children remains scarce.

**Clinical Presentation** A 20-month-old girl presented with torticollis without neurological deficits. Magnetic resonance imaging showed a cavitating mass, with destruction of the C7, T1, and T2 vertebrae. A combined anterior cervical and sternotomy approach was used to rest the tuberculoma, after which an allograft fibula was placed in the gap. An anterior plate was placed. The posterior elements were opened to induce fusion. Neurological status remained unchanged, but after 6 weeks, the allograft was displaced, resulting in kyphosis. This kyphosis remained unchanged, and fusion of the posterior elements developed in the following months.

**Conclusion** Combined anterior cervical and sternotomy approach and additional posterior fusion induction is a feasible surgical strategy for tuberculoma resection of the cervicothoracic spine. In the postoperative course, close monitoring should be performed to detect progressive kyphosis in an early stage.

## Introduction and Importance

Tuberculous spondylitis (TS), also known as Pott’s disease, is a presentation of infection with *Mycobacterium tuberculosis* in the spine. Although the exact incidence of TS is not known, skeletal involvement occurs in up to 1–3% of patients with tuberculosis, and in children, the spine is affected in approximately 50% of patients with skeletal tuberculosis. 1 2 3 4 Up to 90% of the affected vertebrae are located in the lower thoracic or lumbar spine. 5 TS generally destroys the vertebral body and intervertebral disc and can expand to adjacent vertebral bodies. Eventually, collapse of the vertebral body may lead to gibbus formation. 6 If left untreated, the condition may lead to paraplegia, which can occur in the early stage of active infection and also in later stages. 7

Treatment with tuberculostatic drugs is critical, but in the case of spinal cord compression or disease-related deformities, surgical decompression and stabilization may be necessary. However, these surgical procedures can prove to be challenging in very young children, due to age-related anatomy.

In this case report, we present a 20-month-old child with TS of the upper thoracic spine. This case report is reported in line with the Surgical CAse REport (SCARE) criteria. 8

## Case Presentation

The patient was a 20-month-old girl who was originally born in Sudan and migrated to the Netherlands at the age of 17 months. Before her migration, she experienced a period of fever and illness, with transient cervical lymphadenopathy. In our hospital, she presented with a painless torticollis that existed for several weeks, without any acute deterioration.

In the physical examination, no neurological deficits were found. Her head was rotated to the left and backward, and she was incapable of deflecting the head in prone position.

**Fig. 1:**
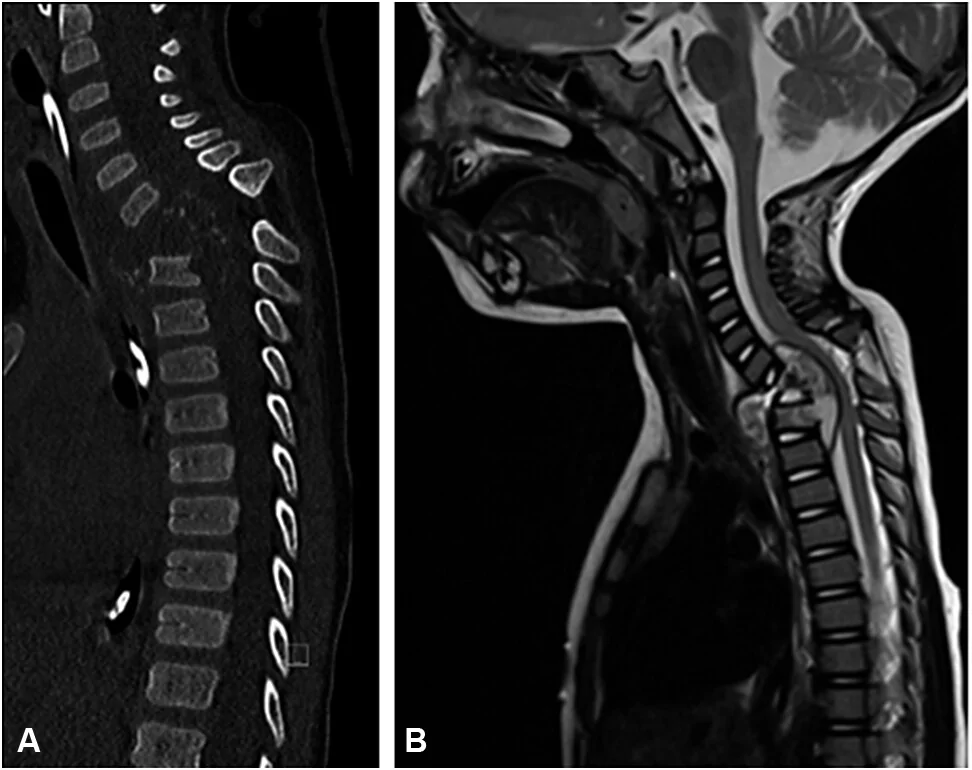
( **A** ) Preoperative computer tomography (CT) of the cervicothoracic spine. Sagittal view. Destruction of C7, T1 and T2 vertebrae with accompanying kyphosis. ( **B** ) Preoperative magnetic resonance imaging (MRI) of the cervicothoracic spine. **B** : Sagittal view. Tuberculoma with destruction of T1 and T2, and a epidural mass with spinal cord compression from C7-T3.

Magnetic resonance imaging (MRI) of the cervicothoracic spine showed a cavitating mass, causing destruction of the corpora of C7, T1, and T2, leading to increased kyphosis. Furthermore, paravertebral and epidural expansion was seen, with Epidural Spinal Cord Compression scale grade 3 from C7 to T3. 9 Computed tomography (CT) of chest and abdomen showed multiple enlarged lymph nodes in the mediastinum but no intrapulmonary cavitating noduli ( Fig. 1 ).

Polymerase chain reaction testing was positive for *M. tuberculosis* , and rifampicin, isoniazid, and pyrazinamide were started. In consultation with the pediatric infectiology, it was deemed preferable to treat with these tuberculostatic agents for at least 1 month before surgery was performed. Therefore, the patient underwent daily neurological examinations to detect eventual neurological deterioration that warranted earlier surgical decompression. In this period, spinal surgery specialists from countries in which tuberculosis is more endemic were consulted to deliberate on the best surgical strategy. Because the vertebral column was considered unstable, patient was treated with a custom-made stiff cervicothoracic brace until surgical decompression and stabilization could be performed. Surgery took place 45 days later; the neurological examinations did not change in this period.

### Surgical Procedure

Halo traction was considered in the preoperative phase to improve spinal alignment. It was decided to refrain from performing Halo traction, because there were concerns about finding proper pin sites, given the young age. Furthermore, and more importantly, the child recently experienced migration to the Netherlands and she and her family were unable to communicate adequately in Dutch or English. Therefore, concerns arose about adequate communication in what would be an impactful procedure.

Patient was transported from her room to the operating room (OR) directly. All personnel wore filtering facepiece-2 masks and protective glasses. Nonsterile personnel wore isolation gowns. The protective measures were in place until 30 minutes after the patient left the OR. Postoperative recovery was performed in the OR, and extubation took place in an isolation room in the pediatric intensive care unit (PICU).

During surgery, intraoperative neuromonitoring (IONM) was performed. The IONM consisted of free running electromyography and transcranial electric stimulation-motor evoked potential of the deltoid, triceps, abductor polllicis brevis, abductor digiti minimi, rectus femoris, tibialis anterior, gastrocnemius medialis, abductor hallucis muscles. Somatosensory evoked potentials were performed bilaterally in the ulnar nerve, median nerve, and tibial nerve.

**Fig. 2:**
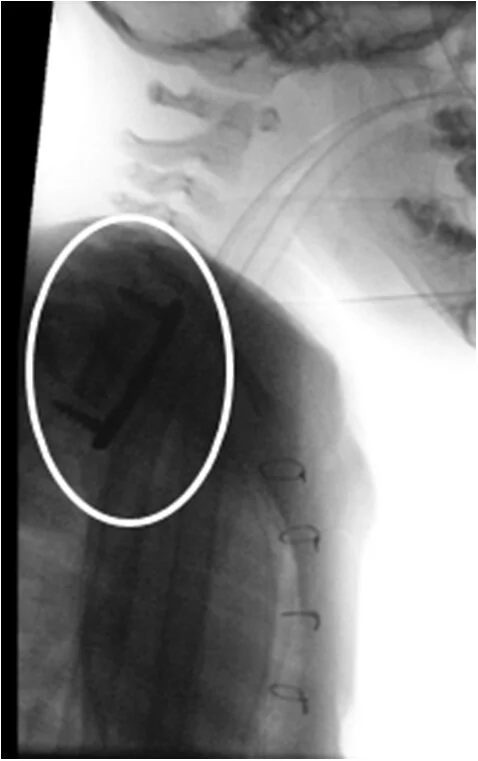
Peroperative imaging after removing the lesion, and placement of an allograft fibula and plate (in the white circle).

The patient was initially positioned in the supine position, because it was deemed sensible to decompress the spinal cord first through resection of the tuberculoma. An anterior cervical approach did not provide adequate exposure of the caudal part of the tuberculoma, and a sternotomy did not provide sufficient exposure of the cranial part. Therefore, we performed a combined anterior cervical approach and a sternotomy. To gain proper exposition, a thymectomy was performed. After identifying the longus colli muscles at the level of C6, the tuberculoma was identified at the levels below. The discs at C6 to C7 and Th2 to Th3 were incised. Then, the capsula of the tuberculoma was incised, and the tuberculoma was evacuated piecemeal. The dura was identified and completely decompressed over the course of the tuberculoma. Given the age-related anatomical dimensions of the patient’s fibula, an autograft fibula did not provide adequate support. Therefore, an allograft fibula was then placed in the gap between C6 and Th3, and a plate was placed ( Fig. 2 ). The incision was closed; postoperative thoracic drainage was applied. The patient was then turned into prone position; on the control imaging, the spinal alignment was unchanged. A midline incision C6 to Th3 was made. Since the diameter of the cervicothoracic pedicles was approximately 1.5 to 2.0 mm, it was not considered feasible to perform posterior instrumentation. Therefore, the cortical bone of the laminae was opened, and bone chips were placed over the trajectory to induce fusion. IONM measurements were stable during the entire surgery. In the direct postoperative course, the patient was transferred to the PICU for close monitoring.

A stiff neck brace was applied to minimize movement of head and neck and therefore help the induction of posterior fusion. In the first 5 days, the patient experienced pain that needed intravenous analgesia. The mobilization period thereafter was somewhat delayed because of kinesiophobia. The neurological examination remained unchanged, without any sequelae. Twelve days after surgery, the patient was discharged home.

### Clinical Course

**Fig. 3:**
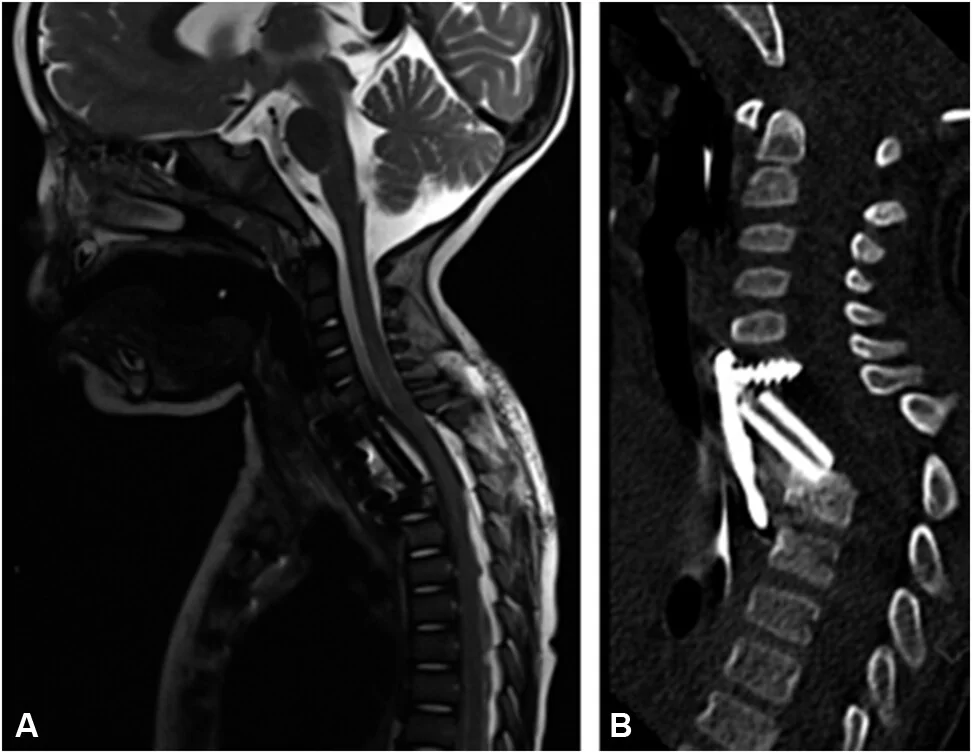
( **A** ) MRI, one week after the surgery. ( **B** ) CT scan 9 months after the surgery.

The patient recovered well from the surgery and did not experience significant side-effects of the tuberculostatic drugs. MRI 1 week after the surgery showed adequate position of the fibula allograft and plate ( Fig. 3A ). Six weeks after the surgery, a routine follow-up CT was performed, showing displacement of the fibula autograft and a changed position of the screws ( Fig. 3B ). There were no accompanying clinical signs. CT did not show fusion at this moment. After multidisciplinary consideration (including neurosurgeons, orthopedic surgeons, trauma surgeons, and radiologists), it was decided to follow-up and continue the stiff brace, to see if fusion could be obtained in a later phase. The patient underwent regular visits to the outpatient clinic, every 2 weeks in the first 6 months, every 4 weeks in the last 3 months. In every 4 weeks, an X-ray of the cervicothoracic spine was performed to assess stability. During the visits, the skin was checked for pressure sores, which she did not develop during the course of the treatment. Approximately 6 months after surgery ( Fig. 2B ), CT showed fusion beginning of the distal part of the fibula autograft, and of the facet joint Th1 to Th2 and Th2 to Th3. No more follow-up X-rays were performed thereafter to limit the radiation burden. Three months later, fusion progressed, and treatment with the stiff brace was stopped. At last follow-up, 17 months after the surgery, the patient experienced no back pain, neurological deficits, or other complaints.

## Clinical Discussion

In this case report, we present a toddler with TS of the upper thoracic spine, treated with anterior corporectomy and allograft fibula through a sternotomy, followed by induction of posterior fusion by opening the cortical bone of facet joints. Due to the destructive nature of tuberculous infections in the spinal column, surgical debridement and stabilization can be a necessary addition to tuberculostatic treatment, especially when deformities arise or when patients have neurological deficits.

Different surgical strategies have been proposed in the literature. For example, Egea-Gámez et al presented two patients of 21- and 23-month-old, respectively, in which they performed a decompression and corporectomy, with anterior structural rib autograft and posterior fixation. 10 One of these patients had lesions in T3 and T4, the other one in T8. Both patients developed kyphosis in the postoperative course, for which the first patient needed revision surgery with a longer trajectory of posterior fixation. Leos-Leija et al presented a case of an 11-month-old patient with TS in T7 and T8, who underwent a decompression and corporectomy and placement of a fibula allograft. 11 On postoperative imaging, the allograft changed position. However, this did not affect neurological functioning. Cavus et al reported a case of a 2-year-old child with TS in T2, T3, and T4, who presented with severe kyphosis and deteriorated neurological functioning after an initial laminectomy. 12 Revision surgery entailed tuberculoma evacuation and posterior fixation with pedicle screws C7 to T7.

As reported in the abovementioned case reports, the transthoracic approach, sometimes combined with costotransversectomy, can be used for anterior corporectomies in mid- or low-thoracic lesions. However, this approach was not suitable for our patient, who had a cervicothoracic lesion. Therefore, we used a combined anterior cervical and sternotomy approach. We refrained from posterior fixation in our patient, because of age-related challenging anatomy and growth potential.

The abovementioned literature shows that treating deformities caused by TS in young children is challenging, and that a variety of surgical strategies are being used. Surgical strategies that are widely used in older children or adults can prove to be difficult due to age-related anatomy. The mechanical properties of bones in children differ considerably from those in adult patients. Pediatric bones are smaller, have reduced mineralization, and contain more collagen, reduced stiffness, and increased ductility and toughness. 13 Also, pedicles in children have less cortical thickness than those in adults, and the synchondroses in pediatric vertebrae tend not to be complete ossified before the age of 3. 14 15 These combination of these factors may have contributed to the fact that all these very young patients undergoing anterior surgery in the literature experienced progression of kyphosis. The safety of cervical and thoracic pedicle screws in the pediatric population has been well-established. However, the children included in these studies are older than our patient, meaning that pedicle sizes are more likely to allow pedicle screw placement. 16 17 Also, the pullout strength increases with age. 18 The pedicle size of our patient was 1.5–2.0 mm. Commercially available pedicle screws normally have a diameter of 3.5–4.5 mm, although some manufacturers also provide screws with a diameter of 3.0 mm. 17

In our patient, the abovementioned processes may also have contributed to the screw displacement in Th3 and therefore to the changed position of the fibula allograft. It might be hypothesized that direct screw fixation of the allograft in the cranial and caudal vertebrae could have helped to maintain its original position. We refrained from doing so to prevent additional damage to the endplates, which in itself could have increased the risk of displacement. Given the fact that bone features in young children are so different from those in adult patients, continued radiological monitoring in these patients is important. Although the ventral displacement of the allograft prolonged follow-up and bracing for our patient, it did not cause other complications or complaints (e.g., dysphagia or local pain).

## Conclusion

Combined anterior cervical and sternotomy approach is feasible for anterior corporectomy with fibula allograft in young children with TS. Due to high risk of progressive kyphosis, close radiological monitoring is important.

## Declaration of GenAI Use

The authors confirm that no Generative AI was used in the preparation of this manuscript.
